# Nucleus preservation in early Ediacaran Weng'an embryo-like fossils, experimental taphonomy of nuclei and implications for reading the eukaryote fossil record

**DOI:** 10.1098/rsfs.2020.0015

**Published:** 2020-06-12

**Authors:** Weichen Sun, Zongjun Yin, John A. Cunningham, Pengju Liu, Maoyan Zhu, Philip C. J. Donoghue

**Affiliations:** 1 State Key Laboratory of Palaeobiology and Stratigraphy, Nanjing Institute of Geology and Palaeontology, Chinese Academy of Sciences, Nanjing 210008, People's Republic of China; 2 Center for Excellence in Life and Paleoenvironment, Chinese Academy of Sciences, Nanjing 210008, People's Republic of China; 3 University of Science and Technology of China, Hefei 230026, People's Republic of China; 4 School of Earth Sciences, University of Bristol, Life Sciences Building, Tyndall Avenue, Bristol BS8 1TQ, UK; 5 Institute of Geology, Chinese Academy of Geological Sciences, Beijing 100043, People's Republic of China; 6 College of Earth and Planetary Sciences, University of Chinese Academy of Sciences, Beijing 100049, People's Republic of China

**Keywords:** Ediacaran, Weng'an Biota, fossil embryo, subcellular structure, taphonomy

## Abstract

The challenge of identifying fossilized organelles has long hampered attempts to interpret the fossil record of early eukaryote evolution. We explore this challenge through experimental taphonomy of nuclei in a living eukaryote and microscale physical and chemical characterization of putative nuclei in embryo-like fossils from the early Ediacaran Weng'an Biota. The fossil nuclei exhibit diverse preservational modes that differ in shape, presence or absence of an inner body and the chemistry of the associated mineralization. The nuclei are not directly fossilized; rather, they manifest as external moulds. Experimental taphonomy of epidermal cells from the common onion (*Allium cepa*) demonstrates that nuclei are more decay resistant than their host cells, generally maintaining their physical dimensions for weeks to months post-mortem, though under some experimental conditions they exhibit shrinkage and/or become shrouded in microbial biofilms. The fossil and experimental evidence may be rationalized in a single taphonomic pathway of selective mineralization of the cell cytoplasm, preserving an external mould of the nucleus that is itself resistant to both decay and mineral replication. Combined, our results provide both a secure identification of the Weng'an nuclei as well as the potential of a fossil record of organelles that might help arbitrate in long-standing debates over the relative and absolute timing of the evolutionary assembly of eukaryote-grade cells.

## 1. Introduction

Interpreting the fossil record of eukaryotes is challenging because their key distinguishing characteristics—organelles and a nucleus—are not commonly preserved. Indeed, where nucleus-like structures occur in fossils, the default interpretation is that they cannot be nuclei (e.g. [1]). In large part, this stems from experiments which show that cytoplasmic shrinkage in decaying bacteria can produce nucleus-like remains [2–4]. However, it does not follow from these experiments that nuclei cannot be preserved and, indeed, there are a number of credible claims of fossilized nuclei [5–14]. Claims of nuclei and even nucleoli preserved in association with embryo-like fossils from the approximately 609 Mya Ediacaran Weng'an Biota [15–20] have proven especially contentious [1,21–23]. These structures exhibit a consistent size and shape and generally occur one per cell except where two occur, bilaterally arranged, interpreted to anticipate the plane of cell division [16]. Rare elongate and dumbbell-shaped structures also occur, interpreted to reflect fossilization during the process of division [19]. The nucleus interpretation has been criticized on the basis that the structures are incomparably large for nuclei and because they occur as late diagenetic, void-filling, botryoidal mineral cements incompatible with exceptional fossilization of nuclei, which are expected to decay rapidly [1,21–23]. Volumetric characterization has shown that, across binary reductive palintomy, the relationship between the size of these structures and their host cells closely matches living model systems; they are not incomparably large [20,24]. However, the style of fossilization that these structures exhibit and the fossilization potential of nuclei remain unclear.

In an attempt to resolve the paradox between the style of preservation exhibited by the nucleus-like structure in Weng'an embryo-like fossils and the anticipated low fossilization potential of nuclei, we undertook a microscale physical and chemical characterization of the variation in the nature of these structures in the fossil, in parallel with an experimental analysis of the decay of nuclei.

## 2. Material and methods

Embryo-like fossils attributable to *Spiralicellula* and *Megasphaera* were collected from the Upper Phosphate Member (or Weng'an Phosphate Member) of the Doushantuo Formation at 54 Quarry in the Weng'an phosphate mining area in Guizhou Province, Southwest China (for further details see [25]). Both *Spiralicellua* and *Megasphaera* are spheroidal and of comparable size (diameters in the range 450–900 µm). *Megasphaera* is characterized by an outer envelope with a cerebral, fractal or dimpled surface ornamentation and palintomic cell division inside the envelope. However, the taxonomy of *Megasphaera* itself is contentious and co-occurring fossils with similar surface ornamentation have been variably attributed to *Megasphaera, Tianzhushania* and *Yinitianzhushania* [25,26]; here, we refer them all to *Megasphaera* pending establishment of criteria on which they may be consistently discriminated. *Spiralicellula* also possesses an ornamented envelope but is distinguished by its spiral cell morphology [26]. Rock samples of grey dolomitic phosphorite were dissolved in *ca*. 7–10% buffered acetic acid [27] and separated from the resulting residues by manual picking under a binocular microscope. Morphological observations were carried out by a combination of X-ray microscopic tomography and scanning electron microscopy (SEM). Energy dispersive X-ray spectroscopy (EDS) elemental mapping and confocal Raman spectroscopy were used for the *in situ* analysis of elements and minerals.

### 2.1. Microscale characterization of fossils

#### 2.1.1. X-ray microscopic tomography

We conducted tomographic analyses using a Carl Zeiss Xradia 520 Versa X-ray tomographic microscope [28] at the Nanjing Institute of Geology and Palaeontology, Chinese Academy of Sciences (NIGPAS), and synchrotron radiation X-ray tomographic microscopy (srXTM; [29,30]) at the X02DA TOMCAT beamline of the Swiss Light Source (SLS; Paul Scherrer Institute, Villigen, Switzerland) and BM5 beamline of the European Synchrotron Radiation Facility (ESRF; Grenoble, France). Measurements on the Xradia instrument were obtained with an operating voltage of 50 kV and 4 W, 4× objective yielding isotropic voxel dimensions of 0.7022–1.3093 μm, LE2 filter, obtaining 3000 projections through a rotation of 360°. srXTM measurements used 10× and 20× objective lenses at SLS (yielding reconstructed tomographic data with voxel dimensions of 0.65 µm and 0.325 µm, respectively) or 10× objective lens at ESRF (voxel dimension of 0.75 µm), at energy levels of 15–20 keV and exposure times of 50–400 ms. About 1501 projections were taken equi-angularly through 180^o^ of rotation within the beam. Projections were post-processed and rearranged into flat- and dark-field-corrected sinograms, and reconstruction was performed on a 60-core Linux PC farm, using a highly optimized routine based on the Fourier transform method and a regridding procedure [31]. Slice data were processed using VGStudioMax (www.volumegraphics.com). X-rays from synchrotron sources are monochromatic and so differences in contrast will reflect the densities of the fossil materials they pass through [32].

#### 2.1.2. SEM

Based on their three-dimensional reconstructions generated by tomography, some specimens were embedded within resins and then cut precisely to expose the well-preserved nucleus-like structures, using diamond wire blade. The nucleus-like structures were then observed using EDS-coupled field emission scanning electron microscopy, Zeiss GeminiSEM 500 at the Demo Laboratory of Zeiss (China, based in Shanghai) and TESCAN MAIA3 at NIGPAS. Both secondary electron and backscattered electron detectors were used for SEM with different accelerating voltages at 5–15 kV. EDS mapping was used at the optimal beam condition (5–10 kV).

#### 2.1.3. Confocal Raman spectroscopy

Confocal Raman spectroscopic analyses were performed on polished surfaces or surfaces of natural fracture, by using a Horiba LabRAM 800HR Evolution coupled with an Olympus microscope at NIGPAS. An air-cooled frequency-doubled Nd:Yag laser with a wavelength of 532 nm was directed through different density filters, with an aperture hole of 100 μm diameter, a 100× or 50× objective, and finally to a minute spot of about 2 μm diameter on the sample with a final power of about 1–5 mW. The data were processed using the software LabSpec (version 6), and the final spectra were subtracted with a linear baseline from 100 to 1700 cm^−1^ to remove the background fluorescence.

### 2.2. Experimental taphonomy

The common onion (*Allium cepa*) was established as an experimental model for studying the taphonomy of cells and organelles by Chen *et al*. [33], who attempted to simulate the fossilization of organelles by silicification. *Allium cepa* is particularly suitable as our experimental organism because the nuclei are readily visualized using standard histological stains and the epidermis can be peeled away in coherent sheets so that large numbers of cells can be easily studied on a single microscope slide. Our experiments do not attempt to recreate the conditions of death, decay and fossilization in a specific environment, organism or developmental stage. Rather, our aim is to obtain general insights into the decay of nuclei under standard experimental conditions to provide a null model against which the preservation of the nucleus-like structures in *Megasphaera* may be compared (cf. [34]).

Layers of epidermis were peeled from fresh *Allium cepa* and cut into *ca*. 10 mm by 10 mm squares, which were then subjected to one of two distinct experimental systems. The first contained tapwater in a sealed container to induce anoxic conditions while the second contained tapwater with 100 mM β-mercaptoethanol (β-ME) in a sealed container to induce reducing conditions. The water was not sterile, and no additional bacterial inoculum was used. These contrasting experimental systems are widely used by many other authors (e.g. [35–39]) who have attempted to isolate the impact of the reducing conditions under which exceptional preservation commonly occurs. Individual pieces of epidermis were recovered from each system using tweezers at 1, 2, 3, 4 and 8 weeks. These were rinsed with water, stained with methylene blue in a Petri dish for *ca*. 45 min and then rinsed in water again. Samples were then flattened on a microscope slide and covered with a coverslip before visualization using light microscopy on a Leica DM LB2 microscope with a Leica DFC450 C colour camera at the Wolfson Bioimaging Facility, University of Bristol, Bristol, UK.

## 3. Results

### 3.1. Microscale characterization of fossil nucleus-like structures

Well-preserved specimens with subcellular structures were identified among hundreds of specimens of *Megasphaera* and *Spiralicellula* characterized using X-ray microscopic tomography. Our data suggest that the cell cytoplasm is always preserved as microcrystalline apatite, while the nucleus-like structures have diverse shapes and mineral compositions. Among these, we identify six principal grades of preservation of the nucleus-like structures.

#### 3.1.1. Irregular outline with apatite inner body and silica shell

**Figure 1.**
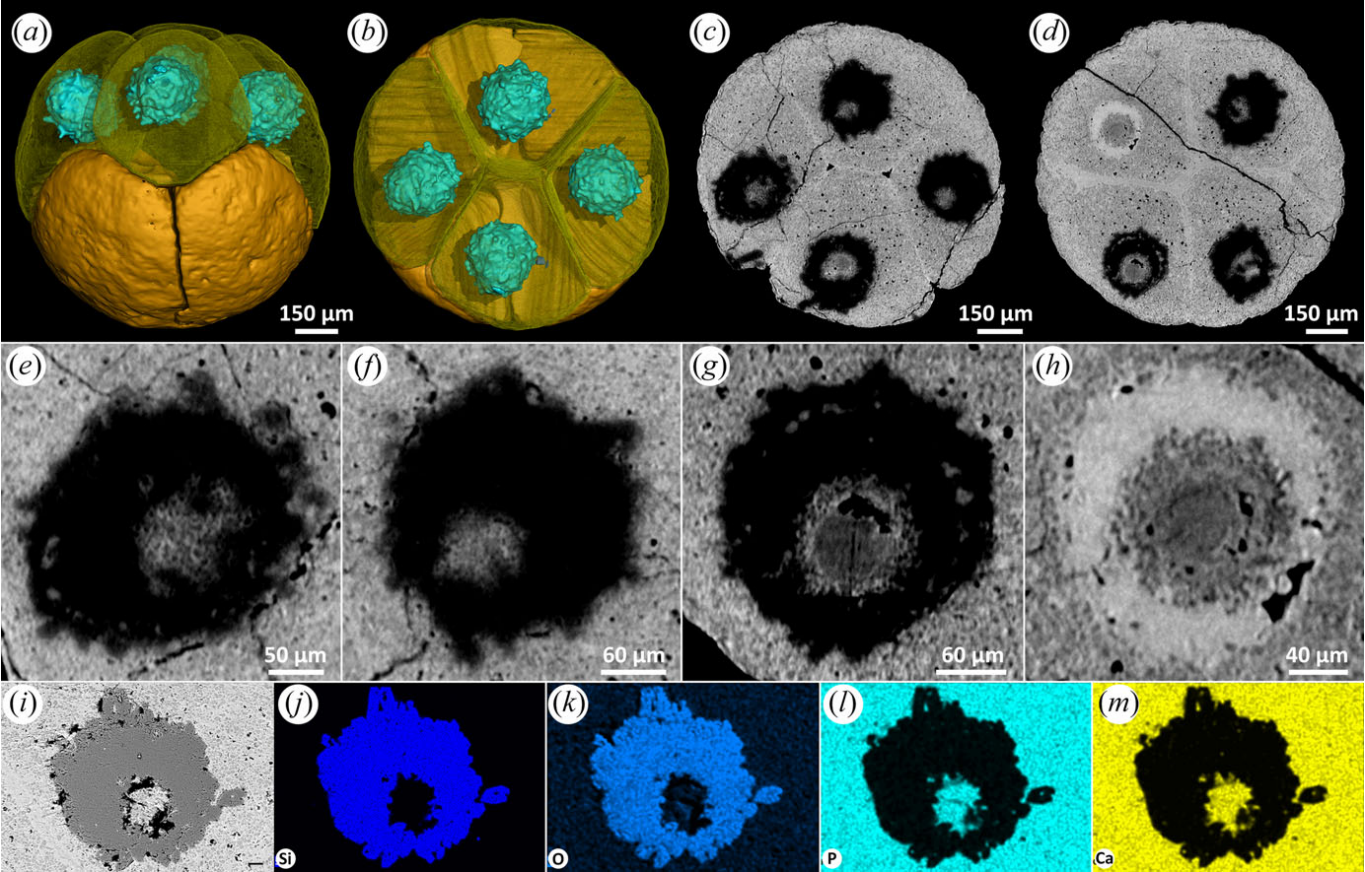
Eight-cell *Megasphaera* from the Ediacaran Weng'an Biota. (*a,b*) Tomographic reconstructions showing nucleus-like structures within cells. (*c,d*) Tomographic virtual sections showing nucleus-like structures. (*e–h*) Magnifications of nucleus-like structures, displaying the details of inner body and shell. (*i*) An SEM image of a nucleus-like structure. (*j–m*) EDS elemental mapping of the nucleus-like structure in (*i*).

The nucleus-like structure is preserved as an outer shell and an inner body in the centre (figure 1*a–d*). The style of mineralization can vary even between cells in a single embryo-like fossil. Figure 1 shows that, in one fossil, the outer shell of one nucleus-like structure is preserved in a high X-ray-attenuating mineral phase (figure 1*d,h*), while the others exhibit low X-ray attenuation (figure 1*c–g*). The inner body has a slightly lower attenuation than the mineralized phase preserving the cytoplasm (figure 1*e–h*). EDS elemental mapping shows that the inner body is characterized by higher relative concentration of Ca and P, consistent with the mineral phase replicating the cytoplasm (figure 1*i,l,m*). The outer shell of the nucleus-like structure, characterized by low X-ray attenuation, exhibits higher relative concentrations of Si and O (figure 1*i–k*), compatible with a chert mineralogy. Notably, the irregular outline of the nucleus-like structures correlates with the homogeneous structure of the cytoplasm.

#### 3.1.2. Irregular outline with homogeneous silica or apatite inner body

**Figure 2.**
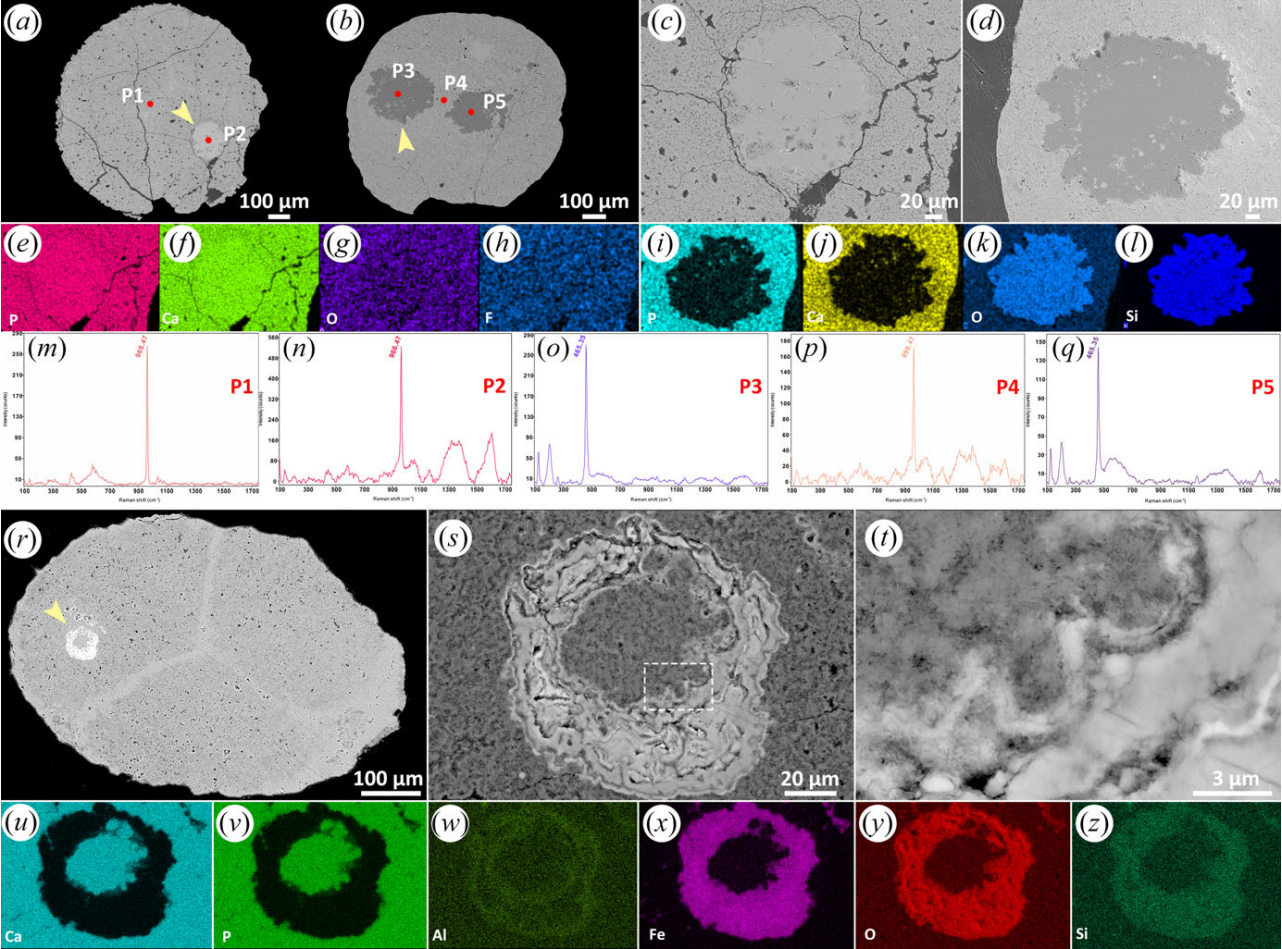
Three specimens of *Megasphaera* from the Ediacaran Weng'an Biota. (*a*,*b*,*r*) Sections showing nucleus-like structures. (*c*,*d*,*s*) Close-up views of the nucleus-like structures marked by arrows in (*a*,*b*,*r*), respectively. (*e–h*) EDS elemental mapping of the nucleus-like structure in (*c*). (*i–l*) EDS elemental mapping of the nucleus-like structure in (*d*). (*m–q*) Raman spectroscopic analyses of the points marked in (*a*) and (*b*). (*t*) Close-up view of the margin of the nucleus-like structure in (*s*). (*u–z*) EDS elemental mapping of the nucleus-like structure in (*s*).

The nucleus-like structures of some specimens exhibit irregular borders with the enveloping cell (e.g. figures 1 and 2). However, although some exhibit heterogeneous mineralization (figure 1), in others the mineralization is homogeneous (figure 2*a*–*l*), though the mineral system can vary between examples. For example, figure 2*a* shows an embryo-like fossil in which the nucleus-like structure exhibits higher X-ray attenuation than the mineral of the enveloping cytoplasm; EDS elemental mapping and Raman point analyses (strong Raman peak at 966.47 cm^−1^) indicate that both are preserved in apatite (figure 2*c,e–h,m,n*). In other specimens the mineral preserving the nucleus-like structure can exhibit lower X-ray attenuation (figure 2*b,d*); EDS elemental mapping shows that the nucleus-like structures are characterized by higher relative concentrations of Si and O (figure 2*i–l*), while Raman point analyses (strong Raman peak at 465.35 cm^−1^) indicate that the nucleus-like structures are mineralized with quartz (figure 2*b,o–q*).

#### 3.1.3. Irregular outline with apatite inner body and iron-rich clay shell

The inner body shown in figure 2*r–t* is irregular in shape and homogeneous in structure and exhibits a similar clotted texture and X-ray attenuation profile to the surrounding cell, with which the nucleus-like structure has an irregular shaped border. EDS elemental mapping shows that the central region and cell cytoplasm are characterized by higher relative concentrations of P and Ca (figure 2*u,v*). A relatively high X-ray mineral phase forms a shell around the inner body with a convolute arrangement of layers characteristic of void-filling centripetal mineralization (figure 2*s*). EDS elemental mapping reveals a composition enriched in Fe, Al, O and Si (figure 2*w–z*), compatible with an iron-rich clay mineral.

#### 3.1.4. Distinct outline with homogeneous apatite inner body

**Figure 3.**
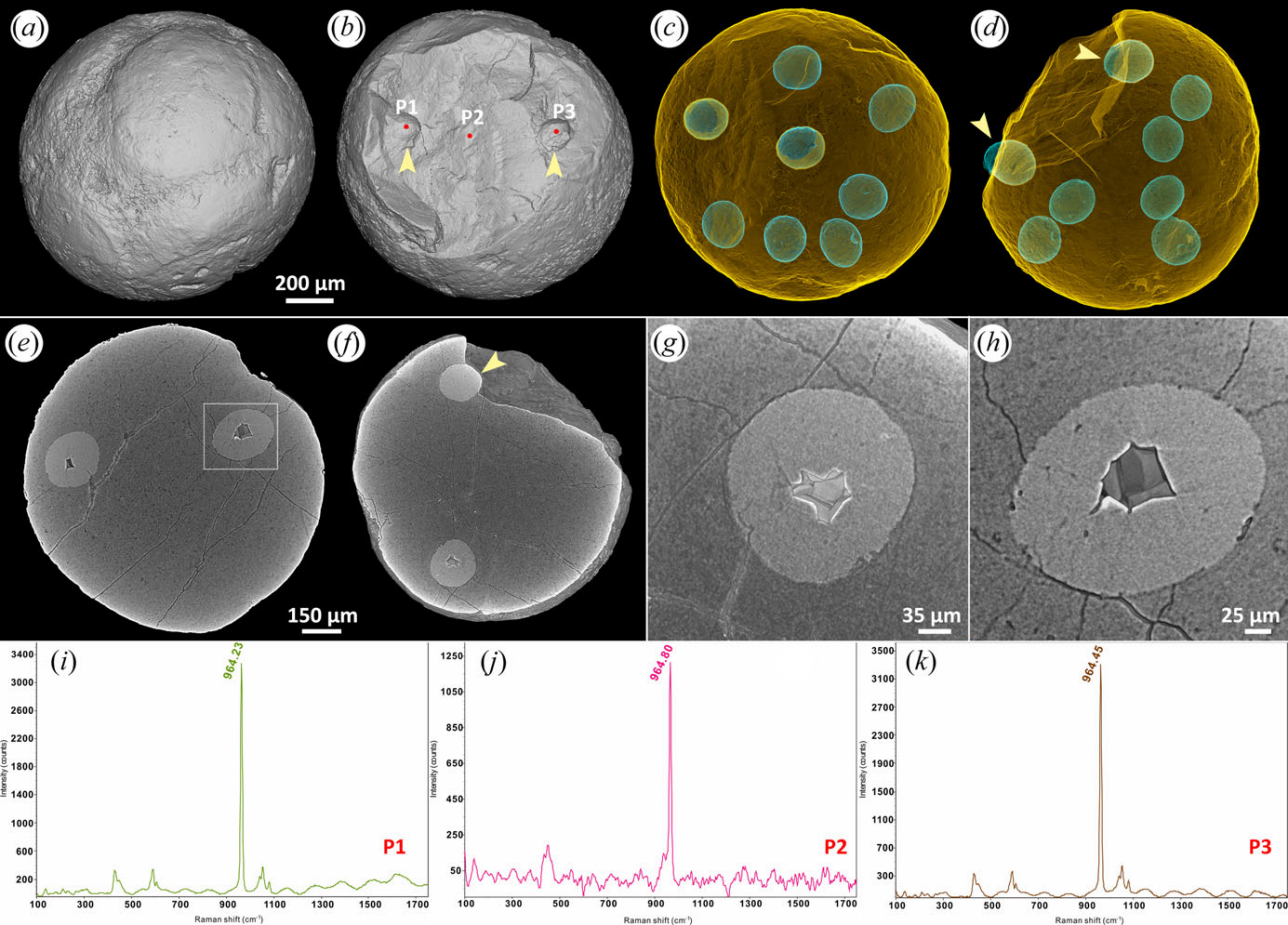
A broken specimen of *Megasphaera* from the Ediacaran Weng'an Biota. (*a,b*) Surface renderings with different angles, showing cells and fracture surface, respectively. (*c,d*) Transparent modes showing eight nucleus-like structures. (*e,f*) Tomographic virtual sections showing nucleus-like structures, with details displayed in (*g,h*). The arrows in (*b,d,f*) indicate the two nucleus-like structures exposed on the fracture surface. (*i–k*) Raman spectroscopic analyses of the points marked in (*b*).

Figure 3 shows an embryo-like fossil of which the multicellular nature is evident from the surface (figure 3*a*) but the component cells are indistinct (figure 3*b–f*). Nevertheless, nucleus-like structures are well defined and of consistent shape and size (figure 3*c,d*; diameters and volumes range from 187.723 to 202.233 μm and from 0.003393 to 0.003914 mm^3^, respectively). All of the nucleus-like structures are preserved in a high X-ray-attenuating mineral phase that exhibits a geode-like prismatic crystal structure compatible with centripetal growth which may or may not completely fill the nucleus-like structure (figure 3*e–h*). Confocal Raman spectroscopy exhibits a strong peak at 964 cm^−1^ (figure 3*i–k*) for the mineral preserving the cells and nucleus-like structures, compatible with apatite. Together, these observations suggest that the higher attenuation of nucleus-like structures results more from crystalline textural variation than from a fundamental compositional difference.

#### 3.1.5. Distinct outline with hollow apatite inner body, clay inner body and shell, apatite lining

**Figure 4.**
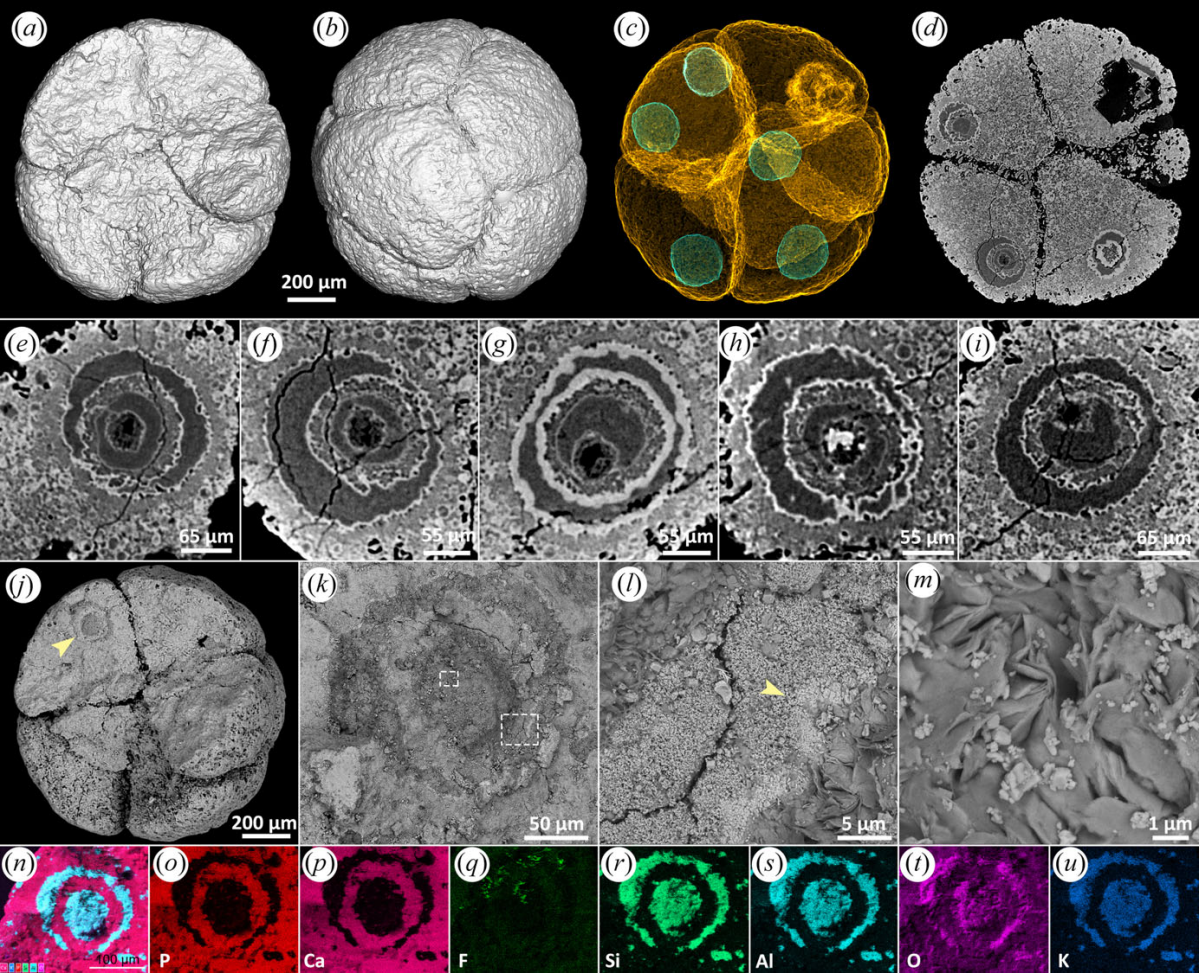
A broken specimen of *Megasphaera* from the Ediacaran Weng'an Biota. (*a,b*) Surface renderings with different angles, showing the fracture surface and cells, respectively. (*c*) Transparent mode showing five nucleus-like structures. (*d*) Tomographic virtual section showing nucleus-like structures, with details displayed in (*e–i*). (*j*) SEM image showing a nucleus-like structure exposed on the fracture surface (arrow), with details displayed in (*k*). (*l*,*m*) Magnifications of the two framed areas in (*k*), showing details of minerals. (*n–u*) EDS elemental mapping of the nucleus-like structure in (*k*).

In some specimens, the nucleus-like structures are distinct from the rest of the cell and are regular and consistent in shape and size, but show multiple concentric shells in mineral phases that exhibit distinct X-ray attenuation profiles (e.g. figure 4). The specimen in figure 4 shows this style of preservation in all but one of the cells. The X-ray attenuation profile alternates between relatively low and high (figure 4*e–i*). The crystal habit in the low-attenuation regions may be fibrous to platy (figure 4*m*), while the high-attenuation mineral phase is microcrystalline, like that of the cytoplasm (figure 4*j–l*). A high-attenuation rim may also define the margins of the nucleus-like structure, associated with larger crystal size (figure 4*l*, arrow). EDS elemental mapping indicates that the low-attenuation mineral phase has higher relative concentrations of Si, Al, O and K, while the higher attenuation mineral phase has higher relative concentrations of P and Ca (figure 4*n–u*). Combined with evidence of crystal habit, we conclude that the high- and low-attenuation mineral phases are apatite and clay, respectively. These main phases may be lined by thinner high-attenuation phases of apatite (figure 4*e–i*) that do not always form complete shells (e.g. figure 4*h*), reflecting centripetal mineral growth within a void space.

#### 3.1.6. Irregular or distinct outline with apatite inner body with an intervening void

**Figure 5.**
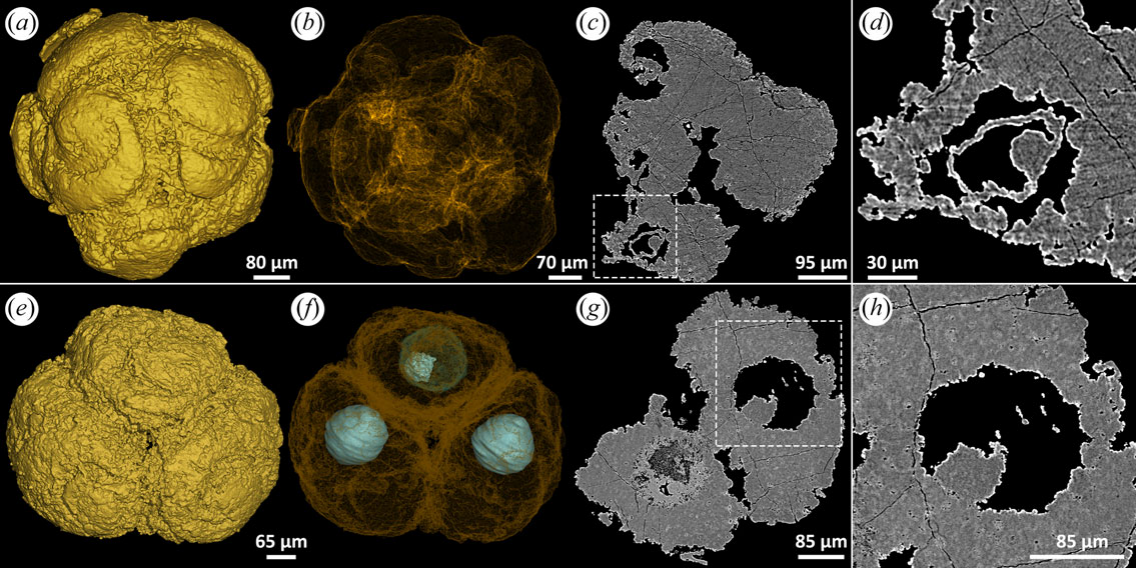
Two specimens of *Spiralicellula* from the Ediacaran Weng'an Biota. (*a,e*) Surface renderings. (*b,f*) Transparent modes of (*a,e*), respectively, showing nucleus-like structures. (*c,g*) Virtual sections of (*a,e*), respectively, showing nucleus-like structures. (*d,h*) Close-up views of the framed areas in (*c,g*), respectively, showing details of the two nucleus-like structures.

The margins of the nucleus-like structure are irregular or distinct while the inner body is preserved in a mineral phase with the same X-ray attenuation profile as the outer cell (figure 5). The inside of the nucleus-like structure is otherwise unmineralized, that is, it is a void space (figure 5*c,d,g,h*). The void space may never have been mineralized or else it may have been filled with calcite or dolomite that was dissolved by the acetic acid used to recover the fossils.

### 3.2. Experimental taphonomy of living nuclei

**Figure 6.**
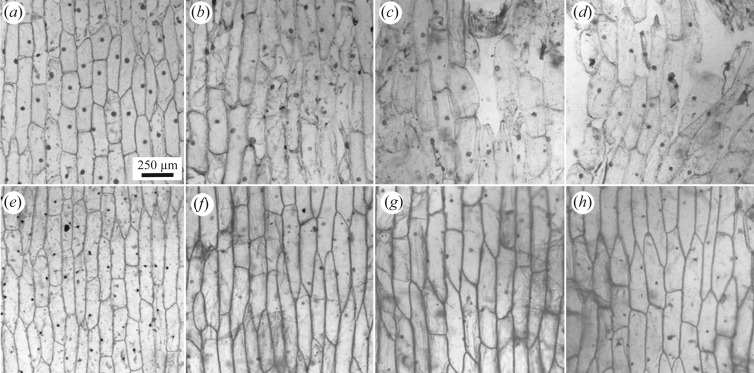
Light microscope images of decayed *Allium cepa* (onion) epidermal cells stained with methylene blue. The specimens in parts *a*–*d* were decayed in reducing conditions for one week (*a*), two weeks (*b*), three weeks (*c*) and four weeks (*d*). The specimens in parts *e*–*h* were decayed in anoxic conditions for one week (*e*), two weeks (*f*), three weeks (*g*) and four weeks (*h*).

After one week, nuclei were still clearly visible in both anoxic and reducing conditions (figure 6*a,e*). In the reducing environment, the nuclei measured approximately 8 µm in diameter, which is close to their dimensions in live material. In anoxic conditions, on the other hand, the nuclei contracted to approximately 50% of their original diameter. After two weeks, the nuclei maintained approximately the size as they did at week 1 in each of the two experimental systems (figure 6*b,f*). However, the intensity of staining diminished, perhaps reflecting biochemical degradation of the nuclei, while maintaining their physical dimensions. Additionally, the cells began to become covered by diffuse material resembling microbial biofilms (figure 6*f*). These were presumably sourced from the water, air or onion surface, given the lack of a bacterial inoculum. While the nuclei remained unchanged at three weeks (figure 6*c,g*), in the reducing system the host cells began to lose adhesion and some cell walls ruptured (figure 6*c*); the cells remained in coherent sheets in the anoxic system (figure 6*g*). After four weeks, the nuclei were still visible in both systems (figure 6*d,h*) and the cells in the reducing system continued to lose adhesion (figure 6*d*). By eight weeks, nuclei were still visible in the cells of the anoxic system, while in the reducing system the cell sheets had lost so much coherence that it was impossible to image them as such.

## 4. Discussion

### 4.1. Taphonomy of the nucleus-like structures in the Weng'an embryo-like fossils

Our microscale characterization of the nucleus-like structures identified three key variables: (i) variation in the shape of the structure which ranged from smooth and consistent to irregular, (ii) the presence or absence of an inner body, and (iii) the mineralogy associated with the nucleus-like structure. The consistency in their size, shape and position [20] indicates that these are homologous structures but variation in their outline shape, from smooth to irregular, and the presence or absence of an inner body appear to indicate that the nucleus-like structures exhibit different degrees of decay prior to mineral replication. Variation in mineralogy suggests that a diversity of taphonomic pathways can be implicated in the fossilization of these structures.

The unaligned microscale structure of the mineral preserving the outer cell contents and inner body (where present) of the nucleus-like structure (e.g. figures 1*g,h*, and 2*s*, 4*e–l*) suggests that they are preserved as a consequence of mineral growth within the biological matrix [17]. Although the inner bodies appear isolated in tomographic sections, they are always physically connected to the outer cell contents at some point, regardless of whether there is an outer shell of distinct mineralization defining the surface of the nucleus-like structure (e.g. figures 4*e,h* and 5*h*). Where an inner body occurs, the outer mineral shell shows clear evidence of being a void-filling cement. This is especially clear in figure 4, where the inner surface of the nucleus-like structures and the outer surface of the inner bodies are lined by a continuous layer of highly X-ray-attenuating apatite, which shows a clear connection where the inner body and outer cell are in contact (e.g. figure 4*h*). Intervening space is filled with a later (younger) clay mineral phase. Void filling is also clear in association with the iron-rich clay mineralization of the shell surrounding the inner body in figure 2*r–t*, evidenced by the palisades of elongate crystallites aligned and arranged in centripetal and centrifugal layers, lining the nucleus-like structure and coating the inner body, respectively, characteristic of late stage mineralization not associated with soft tissue preservation [17].

Where an inner body does not occur within a nucleus-like structure, there remains evidence for void-filling mineralization manifest as large euhedral crystals aligned perpendicular to the inner surface of the nucleus-like structure (e.g. figure 3). Thus, despite the diversity of preservational states and mineral species, the available evidence suggests a common sequence of fossilization in which the cytoplasm and inner bodies undergo mineral replication. The regularity of shape of the nucleus-like structure and the presence or absence of an inner body reflect the degree to which the cells have decayed prior to fossilization. The shape and volume of the nucleus-like structure are preserved as a consequence of non-mineralization, through mineralization of the surrounding cell volume only, producing an external cast of the nucleus-like structure, and then the void formed was later filled by late stage mineralization. This may be rationalized if the nucleus-like structure is decay resistant but not susceptible to mineralization, eventually decaying to leave a void. The diversity of mineral species that perform the role of void-filling cements probably reflects differences in the timing of mineralization during subsequent geological history, and, indeed, the voids are not always filled by cement (figure 5). Inner bodies are often associated with nucleus-like structures whose margins are irregular or poorly defined (e.g. figures 1, 2, 4 and 5). This may indicate some degree of decay prior to mineral replication, in which case the inner bodies may represent shrunken structures that were once the size of the nucleus-like structures.

### 4.2. Experimental taphonomy and fossilization potential of nuclei

Our experimental results indicate that nuclei can survive for at least eight weeks in decay experiments, a time scale that it compatible with fossilization through permineralization [4] as well as microbial biofilm replication and subsequent mineralization [36,37,40]. Indeed, Chen *et al*. [33] have shown experimentally that permineralization of cells and their nuclei in *Allium* can occur in saturated solutions of silica. However, there is some evidence that fossilized nuclei may not faithfully reflect their original dimensions. In our anoxic experimental system, the nuclei shrank during the first week and then maintained approximately the same size for the duration of the experiment. The lack of shrinkage under reducing conditions may be because autolysis is blocked in these conditions [37]. However, in this same system, the cell sheets lost coherence and ruptured; nuclei isolated from their cells would be very difficult to identify even if they do not decay and are fossilized.

It has been argued that nuclei are unlikely to be preserved because of the low preservation potential of the nuclear membrane and that, if nuclei are preserved, we should also anticipate the presence of other organelles that are thought to have higher preservation potential [1]. The argument that nuclei should be preserved alongside other organelles is based on observations of leaves of the Miocene angiosperm *Clarkia*, where nuclei are preserved in lower abundance than chloroplasts and mitochondria [41]. However, the data from *Clarkia* differ from research into senescence in plants, where nuclei are the most stable organelles [42]. It also contrasts with the recent observation that extremely convincing nuclei can be preserved in the absence of other organelles [5]. These findings suggest that the pattern in *Clarkia* may not be generally applicable and that the absence of other organelles is not sufficient to reject a nucleus interpretation. Furthermore, while our experiments do not provide insights into the relative preservation potential of components of the nucleus, they demonstrate that nuclei have a high preservation potential, maintaining their physical dimensions on a time scale compatible with fossilization mechanisms.

### 4.3. Nucleus-like structures in the Weng'an embryo-like fossils are nuclei

Our experiments demonstrate that there is no taphonomic impediment to the interpretation of the nucleus-like structures in the Weng'an embryo-like fossils as nuclei. We have also provided empirical evidence to explain why the absence of other fossil organelles, such as mitochondria and chloroplasts, is not incompatible with the presence of fossilized nuclei. Regardless, mitochondria are difficult enough to observe in living cells without specific stains and contemporary phylogenetic interpretations of the Weng'an embryo-like fossils as holozoans [16,19,23,25,43] is not compatible with the presence of chloroplasts (but see [44]). Nevertheless, our microscale characterization of the nucleus-like structures in the Weng'an embryo-like fossils indicates that they have not been directly fossilized but, rather, they are preserved largely as external casts, through preservation of the surrounding cell volume. This raises the paradox of these nucleus-like structures; if they do represent nuclei and the physical dimensions of nuclei are resistant to decay, why were they not directly fossilized? This reaches to the core of the debate over the explanatory value of taphonomy experiments in attempting to understand the processes of fossilization [34,45].

The preservational variation of nucleus-like structures in the Weng'an embryo-like fossils is, nevertheless, readily rationalized with the results of our decay experiments if the nuclei are not susceptible to mineral replication. Generally, decay resistance and fossilization potential are not inextricably linked because the processes of exceptional fossil preservation are often highly selective and limited to specific tissues, such that exceptionally preserved fossils are not merely the sum of decay-resistant parts; they are often both more and less than this [34]. There is intrinsic evidence that this is the case for the Weng'an nucleus-like structures, our microscale characterization of which demonstrates that the original biological structures were decay resistant, providing a substrate against which an external cast could be preserved during the mineralization of the remaining cell volume. These structures were also not directly fossilized, but the void spaces left after their eventual decay were subsequently filled by late stage diagenetic mineralization long after the loss of original biological substrates or their unmineralized decay products.

However, our analysis of the fossils shows that while in some preservational modes the original biological nucleus-like structures were not directly preserved, in other modes at least some aspects of their anatomy were directly replicated through mineralization, manifest as the inner bodies which have previously been interpreted as nucleoli [19,20]. Notably, our characterization of the styles and nature of mineralization demonstrate not only that the inner bodies were mineralized in the same way and, by inference, at the same time as the surrounding cell volume, but the inner bodies and the outer cell volume are always physically connected through that phase of mineralization. Furthermore, the inner bodies are usually preserved in instances when the nucleus-like structure has an irregular outer surface and shape. Our experiments do not provide any insights into the preservation potential of nucleoli but they do demonstrate that, under anoxic conditions, nuclei shrink and appear to become shrouded in microbial biofilms. Thus, the inner bodies may be more readily rationalized as shrunken nuclei, with the outer dimensions of the nucleus-like structure defined by an unmineralized shroud of microbial biofilm.

Combined, the results of our microscale analysis of the Weng'an nucleus-like structures and experimental taphonomy of nuclei provide for an effective explanation of the fossilization of biological substrates that exhibit a spectrum of decay, preserved via a diversity of mineralization pathways. They also provide the basis for the secure interpretation of the Weng'an nucleus-like structures as nuclei.

### 4.4. Implications for elucidating the fossil record of eukaryotes

Attempts to establish the timing and sequence of assembly of eukaryote-grade cells in the fossil record have been stymied by controversy over the identification of their only definitive characteristic of eukaryotes, the presence of nuclei. Alternatively, researchers have discriminated fossil eukaryote and prokaryote cells based on size, but this diagnosis is probabilistic not definitive [46]. Alternatively, fossil eukaryotic cells are identified on circumstantial evidence of an actin cytoskeleton, such as cyst wall processes or excystment structures which required their cells to change shape in cyst formation or escape, respectively [47]. However, some archaea possess an actin-based cytoskeleton, demonstrating that this feature evolved outside of eukaryotes [48]. Cell or cyst wall differentiation is the only remaining credible criterion for identifying fossil eukaryotes, but even this distinction rests only on the belief that prokaryotes are incapable of such complexity.

A fossil record of subcellular organelles would provide a much more definitive basis for establishing the time scale of eukaryote origin and diversification. This may be complicated by the nucleus-like decay artefacts in bacterial-grade microbes [2–4], but the history of debate over the identification of nuclei in the Weng'an fossils demonstrates that eukaryote organelles can be discriminated based on consistency of shape, size and, for instance, how these change through cell division [19,20]. There are a number of credible examples of nuclei preserved in Phanerozoic fossil eukaryotes [5–14]. Our taphonomy experiments demonstrate the decay resistance of nuclei and the feasibility of their fossilization, both of which are corroborated by our characterization of nuclei in the Proterozoic Weng'an embryo-like fossils.

Thus, we believe that it is time to look again at claims of fossilized nuclei in early Proterozoic and Archaean fossils, hitherto dismissed as possible taphonomic artefacts of prokaryote-grade organisms. This will unleash the power of the fossil record in elucidating the early fossil record of eukaryotes, arbitrating in currently intractable debates over the relative and absolute timing of origin of eukaryote organelles and, ultimately, the emergence of the extant eukaryote diversity.

## 5. Conclusion

We have shown that the controversial nucleus-like structures in the early Ediacaran Weng'an embryo-like fossils exhibit a diversity of preservational modes that differ in terms of their consistency of shape, presence or absence of an inner body and the nature of their mineralization. These structures are not preserved directly; rather, they manifest as external moulds in the mineralization of the remaining cell volume. Taphonomy experiments demonstrate that nuclei are more decay resistant than their host cells, making them available as substrates for mineralization on time scales compatible with exceptional fossil preservation. The fossil and experimental taphonomy evidence is readily rationalized if nuclei are resistant to both decay and mineralization, leaving behind nucleus-shaped voids after their eventual degradation on geological time scales, voids that are later filled by diagenetic mineralization. Together, our results demonstrate not only that nuclei are decay resistant, but also that they can be fossilized and preserved even on ancient geological time scales. Thus, there is the real possibility of a fossil record of organelles that might elucidate the evolutionary assembly of the eukaryote-grade cells.

## Data accessibility

All the specimens cited in this paper have been deposited in NIGPAS and the tomographic data arising from our study are available from the University of Bristol Research Data Storage Facility (http://dx.doi.org/10.5523/bris.2v3sw3xjkaum724prs1ziow4u0 and https://doi.org/10.5523/bris.pxup7vdmg25r2sl3kr00y5cu6).
